# Leveraging AI to Accelerate Competency-Based Education in the Health Professions

**DOI:** 10.5334/pme.2594

**Published:** 2026-10-22

**Authors:** Kimberly D. Lomis, Martin V. Pusic, Eric Warm, Jamiu O. Busari, Soren Huwendiek, Ian W. Incoll, Lydia Siew Tiang Lau, Christy K. Boscardin, Jonathan H. Chen, Pedro Tanaka, Karen E. Hauer, As members of the International Competency-Based Health Professions Educators Collaborative

**Affiliations:** 1 Josiah Macy Jr. Foundation, New York, NY, USA; 2 American Board of Medical Specialties Research and Education Foundation, Associate Professor of Pediatrics and Emergency Medicine, Harvard Medical School and Scholar-in-Residence, Brigham Education Institute, Boston, MA, USA; 3 University of Cincinnati College of Medicine, Cincinnati OH, USA; 4 Faculty of Health, Medicine, and Life Sciences, Maastricht University, The Netherlands; 5 Horacio Oduber Hospital, Aruba; 6 Department for Assessment and Evaluation, Institute for Medical Education, University of Bern, Switzerland; 7 Graduate Programs in Surgical Education, University of Melbourne Melbourne, Australia; 8 Faculty of Health and Medicine, University of Newcastle, Gosford, New South Wales, Australia; 9 Alice Lee Centre for Nursing Studies, National University of Singapore, Singapore; 10 University of California, San Francisco (UCSF) School of Medicine, San Francisco, CA, USA; 11 Academic Office, Taipei Medical University and Hospital, Taipei, Taiwan; 12 Stanford University School of Medicine, Stanford, CA, USA; 13 Department of Medicine, University of California, San Francisco (UCSF) School of Medicine, San Francisco, CA, USA

## Abstract

The concurrent uptake of both artificial intelligence (AI) and competency-based health professions education (CBHPE) represents a watershed opportunity to improve and streamline educational processes and enhance educational effectiveness. Members of the International Competency-based Health Professions Educators Collaborative (ICBHPE) explored the potential of AI to strengthen CBHPE implementation by addressing persistent challenges in meeting its five core components: defined outcome competencies; progressive sequencing; tailored learning experiences; competency-focused instruction; and programmatic assessment. Effective integration of AI tools can be guided by socio-technical systems theory (STST), which highlights the significant interdependence of technical systems and social systems. Successful application of AI in HPE will hinge on addressing the complex interactions among people and roles; goals and incentives; workflows and processes; technology; infrastructure; and organizational culture. Health professions educators should enhance their fundamental knowledge of how AI tools are developed and deployed to understand both its potential and its limitations. The HPE community must rigorously evaluate AI applications to ensure responsible and equitable integration that complements human relationships and judgment.

## Background

The concurrent uptake of both artificial intelligence (AI) and competency-based health professions education (CBHPE) represents a watershed opportunity to improve and streamline educational processes and enhance educational effectiveness. HPE programs must prepare learners and their teachers for an AI-enabled and transformed clinical environment that does not yet fully exist [1,2,3]. Such preparation demands more than simply adding AI content or AI-driven process improvements to existing curricula; it requires a fundamental reimagining of how competency is defined, developed, demonstrated, and assessed in an AI-enhanced healthcare landscape.

To address the dynamic needs of preparing professionals for modern healthcare practice, HPE teachers and leaders have re-examined the limitations of traditional educational structures, such as classroom-based learning and rigid, time-based advancement [4]. CBHPE represents a promising solution to reform the education system, emphasizing explicit demonstration of learning outcomes, personalized and flexible learning pathways, learner agency in developing and demonstrating competency, and authentic assessments in real-world contexts [5]. This adaptive model views competency as demonstrated through meaningful engagement in team-based clinical care with contextualized learning experiences rather than through standardized delivery of predetermined content. However, CBHPE implementation has been hampered by multiple challenges, including learner and supervisor perceptions of assessment burden, insufficient feedback quantity and quality to support learning, inability to synthesize large amounts of learner performance data efficiently, and challenges in customizing future learning [6,7,8,9].

During their 2025 forum, members of the International Competency-based Health Professions Educators Collaborative (ICBHPE) explored the potential of AI to strengthen CBHPE implementation and address these persistent challenges. In summarizing the ICBHPE deliberations, the authors of this manuscript apply the lens of sociotechnical systems theory, elaborated below, to describe how integrating AI into HPE can strengthen the implementation of the core components of CBHPE [10] (Table 1).

**Table 1:** Affordances of big data and AI to support CBHPE core components.


CBHPE CORE COMPONENT	DESCRIPTION	IMPACT OF BIG DATA	IMPACT OF AI TOOLS	PROMOTING FAIRNESS

**1. Outcome Competencies**	Clearly defined competencies expected of all graduates; anchors curriculum to societal and professional needs.	Aggregated learner and patient outcome data can validate or recalibrate competency frameworks across institutions; risk of over-standardization.	AI can map competencies dynamically to clinical tasks and personalize competency attainment pathways. Risk of privileging AI-derived outcomes not aligned with professional judgment.	Ensure competencies reflect diverse populations and approaches. Avoid embedding biased outcome metrics that systematically disadvantage certain groups of learners.

**2. Sequenced Progression**	Learners advance based on demonstrated competence rather than time; progression is staged and developmental.	Longitudinal data supports fine-grained tracking of progression; may highlight hidden inequities in learner advancement.	AI can predict readiness for advancement, flag struggling learners earlier, and tailor pacing. Risks of over-reliance on opaque algorithms.	Guard against data-driven premature labelling of individuals into developmental tracks, or acceleration favoring learners with more digital trace data (e.g., those benefitting from social capital).

**3. Tailored Learning Experiences**	Training adapts to learner needs, clinical context, and patient populations; flexible and personalized.	Big data enables benchmarking of exposure opportunities across cohorts and sites; identifies gaps in case mix or learning opportunities.	AI-driven adaptive learning platforms can recommend individualized content and simulate rare clinical scenarios.	Ensure all learners have adequate technology infrastructure to benefit from personalized experiences. Recognize risk that those serving in resourced-constrained settings may not be exposed to clinical AI tools.

**4. Competency-Focused Instruction**	Teaching is deliberately aligned with competencies; emphasizes coaching and feedback.	Analytics can reveal patterns in feedback quality and instructional practices across faculty; risks of surveillance culture.	AI can support just-in-time feedback (e.g., natural language processing of clinical notes or simulations). Danger of feedback being reduced to automated metrics.	Access to high-quality coaching must be maintained for all learners; AI tools should augment, not replace, interaction with human educators.

**5. Programmatic Assessment**	Multiple low-stakes data points aggregated for high-stakes decisions by committees.	Big data enables integration of thousands of assessment data points to increase reliability; risks of information overload and data misuse.	AI can synthesize multimodal assessment data into actionable insights; can support CCCs in decision-making.	Risk that AI-supported assessments may have linguistic or cultural errors. Establish processes for algorithmic vigilance. Maintain human oversight of high-stakes decisions to protect learners from potential systemic disadvantage.


This manuscript provides evidence-informed, practical strategies for integrating AI into CBHPE across institutions with varying levels of resources and technological readiness, anchored in a sociotechnical systems theoretical framework. In the face of rapidly evolving AI capabilities, we provide a roadmap for responsible experimentation and iteration while maintaining focus on the fundamental principles that make CBHPE transformative.

### AI and the 5 Core Components of CBHPE

Implementing CBHPE is challenging, and incomplete approaches may not produce desired results. The core components framework was developed to guide and evaluate the fidelity of CBHPE implementation, which entails designing and delivering CBHPE as intended and adhering to its core principles and components. Attention to fidelity can avoid implementation error: “*attributing negative findings to a failure in program theory when negative findings may actually reflect an error in program implementation*” [10]. The five core components – defined outcome competencies; progressive sequencing; tailored learning experiences; competency-focused instruction; and programmatic assessment – can be applied proactively to facilitate comprehensive CBHPE implementation [11] that considers and leverages AI’s influence on practice and learning. Examples of robust, cutting-edge AI deployments are provided throughout this manuscript. The affordances of AI can advance the core components summarized in Table 1, and accessible ideas for educators to experiment with publicly available large language models (LLMs) are presented in Table 2.

**Table 2:** Using publicly available large language models (LLMs) to support CBHPE core components.


TRY THIS AT HOME

**Clearly defined outcomes for CBHPE** LLMs can be used to generate “competency maps” from authentic learner work to clarify what skills are truly being used in practice. A program can upload de-identified clinical notes, sign-out texts, consult requests, or procedure summaries and have the LLM analyze them for individual or group patterns in skills such as reasoning, communication, data gathering, efficiency, and adaptability.

**Sequenced Progression** Use an LLM to generate individualized “next best learning experiences” for each trainee. The program can input de-identified information about recent clinical tasks, case mix, simulation performance, and areas of struggle. The LLM can then recommend: what types of cases the learner needs next whether they require more repetition or complexity and which learning environments would best support their developmental readiness.

**Tailored Instruction** Empower trainees to generate their own personalized learning modules. Learners can ask the LLM to create: cases matched to their level of complexity tolerance exercises targeting specific subskills scripts for upcoming difficult conversations time-pressured triage simulations This fosters agency, mastery learning, and adaptive expertise.

**Competency Focused Instruction** Use LLM to extract “instructional snapshots” from daily work so faculty can deliver high-impact micro-teaching. By pasting de-identified notes, sign-outs, or order sets into LLM, a faculty member can ask: identify 1–2 high-value teaching targets for this learner based on their recent work. surface actionable items (e.g., inconsistent problem representation, unclear anticipatory guidance), enabling precise, efficient teaching.

**Programmatic Assessment** Use an LLM to tag faculty narrative feedback to local EPAs or milestones—instantly expanding narrative data relevance. Faculty or coordinators can input batches of de-identified narrative comments and ask the LLM to: tag each comment to the relevant competency domain identify whether the comment is formative, summative, or reflective detect patterns across rotations or teams With careful guardrails, LLMs can be used to monitor for quality and consistency. LLMs can summarize narrative comments and case logs across a cohort, to help program leaders identify whether any learners receive less or fundamentally different feedback, fewer procedural opportunities, or fewer high-acuity cases, prompting corrective action. This dramatically increases the frequency, quality, and usability of narrative assessment, the backbone of programmatic assessment.


#### Clearly Defined Outcome Competencies

##### Current state

CBHPE emphasizes alignment of learning outcomes with the needs of patients and society [12], shifting the concept of competencies from a static set of skills or a fixed curriculum to a dynamic framework responsive to evolving clinical practice. Traditional constraints on assessment and a lack of agility in providing customized training opportunities have historically forced educational programs to focus on more simplified approaches to competency, driven largely by what is convenient to measure [13]. This leads to an overemphasis on some domains of competency, such as medical knowledge, with a lack of attention to other, equally valuable domains and emerging competency demands [14].

##### Potential contribution of AI

AI capabilities facilitate an evolving view of competency as a relational process in which data (capturing clinical cases, outcomes, and patient-reported feedback) and the environment (AI-augmented healthcare) intersect. Competency attainment in an AI-facilitated environment relies on strengthening connections among related skills and knowledge domains and nurturing each professional’s adaptability to evolving demands [15]. As AI and big data are utilized to analyze the relationship between educational interventions and clinical outcomes, educators are likely to identify impactful competencies that are currently under-appreciated. Such an expanded, data-driven perspective on good performance would enhance transparency regarding desired learning outcomes and could better acknowledge the strengths of learners across diverse backgrounds [16]. Aggregate performance data within a given discipline can provide insights regarding existing competency frameworks (e.g., entrustable professional activities (EPAs), milestones) to determine their fit with what practice actually looks like in the real world. This refined alignment would enhance learners’ trust in assessment by anchoring it in the shared value of optimizing patient care [17]. Finally, AI literacy itself becomes an integral core competency for both educators and trainees that will impact all other domains.

##### Application

One example of leveraging AI to recognize competency in new ways is found in the realm of surgical simulation and practice. Researchers are now collecting “thick” multimodal data, which involves using sensors to capture motion, pressure exerted on tissues, and surgeon biometrics and aligning this with video of the procedure [18]. These rich data provide insights into procedural expertise that are not readily apparent to a human supervisor, thereby defining new elements of skills-based competency. Beyond the core component of defining outcomes-based competencies, this advance also benefits other core components, such as the provision of tailored learning experiences. It can achieve this via AI-enabled processing, integrating sensor data in real time to provide a proceduralist with specific, actionable feedback to continually enhance technique.

Another application opportunity for defining outcomes-based competencies is that HPE has traditionally defined competency at the individual level; however, healthcare is delivered in teams. AI is being used to facilitate the capture and analysis of complex interactions between team members, individuals’ use of informational tools, and rich contextual variables such as clinical setting and patient comorbidities [19,20]. Educators will be positioned to examine concepts such as collective intelligence and distributed cognition, which shape an individual’s reasoning and performance in real clinical workflows [21,22,23]. This will shed new light on core competencies for success in real workflows and enable educators to embrace the complexity of the working-learning environment in ways that were not possible before.

#### Learning is Sequenced Progressively

##### Current state

Competencies are ideally organized to support progression while allowing learners to advance at variable rates. In reality, the administrative complexity of managing learner cohorts with limited clinical placement options and rigid timelines, coupled with the need to meet clinical service requirements, currently results in a haphazard sequencing of clinical experiences. Each learner has exposure to a different mix and sequence of learning opportunities, based not on pedagogical logic but on variations in service assignments, supervisory teams, and patients’ presenting complaints. Such variation in experiential sequencing has been demonstrated to impact assessment outcomes negatively [24].

##### Potential contribution of AI

Applying AI tools to study learning outcomes across various sequences of clinical exposures could help educators optimize sequencing. Predictive learner modeling leverages AI to forecast each learner’s likely progression, flagging those who may advance early or require additional support, thereby enhancing responsiveness to individual needs [10,25,26,27]. AI enables dynamic sequencing that aligns educational and health care delivery goals with minimal administrative burden. For example, AI could couple available schedule data with learner data to identify potential “service-neutral” swaps that advantage learners while still supporting service coverage needs.

The ability to synthesize multi-source observations (direct observations, in-training exams, simulation scores) over time could generate recommendations for progressive autonomy, in which learners’ increasing competence is matched with increasing entrustment [28,29].

##### Application

Surgical programs collaborating in the implementation of a specialty-driven EPA framework used a smartphone-based application to capture frequent workplace-based assessments [30,31,32]. Applying data analytics across a greater volume of assessments provides insight into each resident’s progression toward unsupervised practice. The zone of proximal development is the space between what individuals can perform independently and what they can perform with scaffolding or support from a supervisor or colleague (“a more knowledgeable other”) [33,34]. Real-time insights about individual performance offer more precision in aligning each learner’s experiences within their zone of proximal development [35,36]. Future applications of such smartphone-based technology could provide nudges that alert faculty when a resident has accumulated sufficient assessment evidence to justify progression to greater autonomy in a particular task.

#### Tailored learning experiences

##### Current state

To optimize competency development, CBHPE seeks to individualize learning experiences through varied, contextually appropriate educational formats, clinical encounters, and tasks. It has long been recognized that providing individualized support can improve a learner’s performance by two standard deviations [37]. Such support has heretofore been untenable due to resource constraints, with learners lamenting a lack of specific feedback and targeted coaching.

##### Potential contribution of AI

The concept of precision education [26,36] espouses leveraging data and technology to make learning more personalized, efficient, and aligned with authentic work. Precision education systems enhance learner agency over one’s own development, fostering master adaptive learners [15] positioned to thrive in a rapidly evolving health care environment. Capabilities of AI help make such systems feasible by facilitating the integration of multiple data streams about performance (both educational and clinical) to generate actionable learning analytics. Large language models can support an educational program’s responsiveness to such data and its ability to deliver tailored learning experiences. Generative AI also empowers learners to modify existing educational resources into formats that they prefer. Such curricular agility could combat the systemic disadvantages that many learners encounter [38].

##### Application

AI tools can generate and deliver interactive individualized clinical case simulations and provide instant feedback. This enables learners to engage in deliberate practice around a specific learning need at a scale previously unattainable through structured active learning sessions or traditional simulation [39]. LLMs can inform customization of learning experiences that consider the learner’s prior knowledge, skills, experiences, and developmental trajectory.

AI can also be deployed to conduct electronic health record (EHR)-based audits of clinical work activities to track exposure to intended learning experiences and provide tailored feedback, such as evaluating clinical reasoning from EHR notes [40].

AI-enabled dashboards can help trainees recognize gaps in experience and exposure and identify areas where their diagnostic capabilities are strong versus those that need more practice. Such information can guide strategic patient assignments aligned with each learner’s needs and even “nudges” to learners, elevating relevant educational resources in the moment to support tailored learning.

#### Competency-focused instruction

##### Current state

CBHPE teaching practices are ideally linked to desired competencies. In some cases, educators lack insight into the desired competency outcomes and each learner’s current levels of competence [6,41]. Learners and educators alike have been wary of formal educational handovers due to a lack of clear metrics and communication methods [42,43]. Complicating this, supervisors in clinical learning environments are asked to address both patient and learner needs simultaneously. These circumstances make it challenging to deliver competency-focused instruction while navigating a health system oriented toward high throughput and brief interactions (both clinical and educational).

##### Potential contribution of AI

AI-supported tools such as dashboards and goal-setting platforms could help educators, particularly clinical supervisors who do not necessarily identify primarily as educators, focus their instruction on a given learner’s needs efficiently, reducing tensions between the educational and care-delivery missions and making the most of limited bandwidth. Helping educators consider competency in the context of clinical care by providing real-time insights to both the learner’s gaps and the educator’s understanding of desired learning outcomes would align instruction and workflow. If AI and data enable the program to support every learner in pursuing an individualized learning plan, the traditional view of remediation is supplanted by a culture of continuous improvement. For individuals who require more developmental support, more comprehensive data would inform targeted, competency-focused interventions.

##### Application

AI tools are also being developed to analyze the “digital data trails” of authentic daily activity, leveraging routine work artifacts to enable educational nudges and just-in-time feedback [44]. This information can empower learner agency in competency development, supporting both this CBHPE core component and the component on tailored learning experiences.

Integration of clinical and educational data streams offers the opportunity to track patient outcomes as performance indicators; this alignment of learning interventions and patient outcomes can identify important dimensions of performance that are currently under-assessed. Educators have long sought to link patient outcomes to educational interventions. Resident-sensitive quality measures (RSQMs) seek to advance that association [45] but were initially developed as proof of concept and were not meant to be used in real time. Building on RSQMs, TRainee Attributable & Automatable Care Evaluations in Real-time (TRACERs) [46] leverages AI to expedite the link between action and outcome. Supervisors are provided timely insights to anchor their instruction in the competencies directly relevant to the patients the trainee is striving to serve.

#### Programmatic assessment

##### Current state

In CBHPE, learner assessment is organized as an integrated system that aggregates multiple and varied data points over time to support defensible decisions about learner progression [47,48]. Programmatic assessment demands multisource performance evaluations that probe diverse dimensions of competency using multiple assessment methodologies to align with the desired learning outcomes. Although HPE unfolds in a data-rich environment, data are heterogeneous and often reside in siloed platforms, and HPE programs often lack the data deemed most useful for decision-making [24,49].

##### Potential contribution of AI

Educators can leverage AI capabilities to integrate different performance measures that describe a given competency. LLM-based sorting and analysis can enable greater use of narrative data, for example by tagging free-text comments with competencies, milestones, or other relevant frameworks.

Acknowledging that an overall assessment program is limited by the program’s ability to capture performance in a busy work environment, there is active exploration of how AI can complement direct human observation. For example, using ambient technology to capture and analyze trainee-patient interactions offers an alternative form of observation. This enables greater attention to domains of competency for which feedback is commonly limited, such as communication skills and clinical reasoning [27,50,51].

##### Application

Successful use of AI to support programmatic assessment is described in a case study by one internal medicine residency program [52,53]. Program leadership defined observable practice activities for each rotation to focus assessment efforts. Technical integration of diverse data sources, including entrustment ratings, multisource feedback, clinical outcomes, self-assessment, and EHR-derived efficiency metrics, enabled longitudinal programmatic assessment. AI techniques were applied, while maintaining human oversight, to highlight developmental trends, suggest targeted resources, and map learning trajectories to support promotion decisions.

### Summary of Core Components and AI

AI will catalyze implementing CBHPE with fidelity, enabling educators to realize the core components to degrees previously difficult to attain and facilitating more personalized and relevant experiences for learners. The educational use cases for AI are rapidly expanding as both educators and learners explore the possibilities [27,39,54]. The strategies described here are a gateway to a future in which learning and work are tightly integrated seamlessly. AI-enhanced CBHPE may prove a data-driven equalizer of opportunities to learn and advance through training [55]. Attention to organizational culture will be critical to creating an environment in which AI tools complement human relationships and judgment, supporting continuous competency growth for all team members.

### Successfully integrating AI to support CBHPE: socio-technical systems theory (STST)

Although AI holds promise for accelerating the implementation of CBHPE, technology alone will not ensure transformation [4]. The introduction of the EHR in the United States offers a cautionary tale. A technology intended to facilitate clinical documentation became increasingly focused on billing and reimbursement processes [56,57]. The resulting detrimental impact on workflows and increasing burnout among health professionals demonstrates that insufficient attention to the context in which a technology is applied can have significant unintended consequences [58].

Socio-technical systems theory (STST) is a framework that can assist educators in addressing the many variables that influence the successful integration of AI. An approach often used by the AI developers with whom educators will collaborate, STST is one of several sociomaterial frameworks that promote a broadened perspective in HPE research and practice, considering the human, systems, and technological factors that influence behaviors and outcomes [59].

STST asserts that technical and social systems are highly interdependent and that the successful application of emerging technology hinges on addressing the complex interactions between them [60,61]. Key elements that must be addressed when using AI to enable CBHPE include: 1) people and roles, 2) goals and incentives, 3) workflows and processes, 4) technology, 5) infrastructure, and 6) organizational culture (Figure 1).

**Figure 1:**
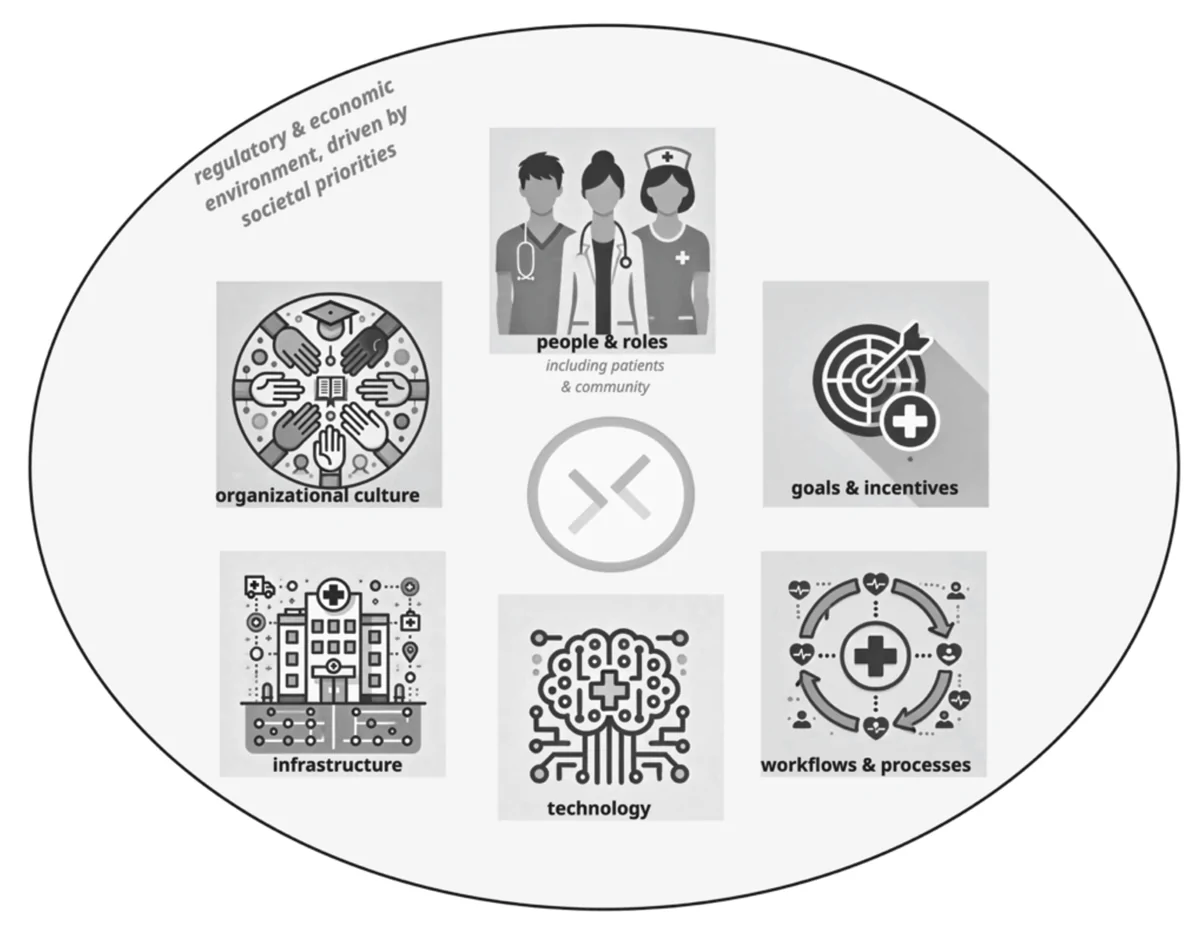
Socio-technical systems theory. Socio-technical systems theory describes the complex interactions between people & roles; goals & incentives; workflows & processes; technology; infrastructure; and organizational culture, all in the context of the broader environment. Fully optimizing the power of artificial intelligence in care delivery and in health professions education requires consideration of all these elements. (*images generated with DALL-E*).

AI offers the potential to bridge educational and health systems to drive CBHPE. Viewed through STST, these intersecting systems are multi-faceted. They hire and recruit **people** (clinical professionals, administrators, educators, researchers, support staff, students and trainees) who fill various **roles**. Collaborating with one another, as well as with patients, families, and communities, these people work toward the common **goals** of providing care while concomitantly training the workforce. People are influenced by individual **incentives** (such as reimbursement, quality metrics and educational advancement). These people intersect through complex **workflows and processes** (both clinical and educational), facilitated by **technology** (diagnostic and therapeutic equipment, informatics systems, and, increasingly, AI). Institutional **infrastructure** (physical and virtual workspaces, computing capabilities, energy sources) and **organizational culture** (social norms and customs) underpin these interactions. This complex, interdependent socio-technical system unfolds within a broader environment (regulatory, legal, and ethical considerations at the macrosystem level) and is driven by societal needs and priorities.

#### Applying STST in the Use of AI to Advance CBHPE

STST is a useful lens for leveraging the power of AI to advance CBHPE. STST reinforces that **people** (both educators and learners) need training in the use and limitations of AI. Educators should consider how **roles** will be modified in an AI-enabled health system, prompting adjustment of necessary competencies. Health professionals are shifting from stewards of knowledge to an augmented-intelligence approach, in which expert interaction with AI and other resources enhances access to and application of knowledge. Such future visioning of evolving roles should inform realignment of health professions training, recruitment and selection, and efforts that support professional identity formation [62,63]. Roles and the professional identity of educators themselves will also continue to shift from delivering content toward fostering master adaptive learners, who learn, grow and adapt in response to the people and systems around them [64]. **Incentives** in education are closely tied to assessment programs, so those programs must be redesigned to elevate skills in information retrieval, critical appraisal, and application. Clinical and educational **workflows** must be adjusted to allow time and space for the meaningful integration of AI insights about competency. Educational **technology** must be developed and deployed in responsible ways, with input from educators and learners into the design, implementation, and ongoing monitoring of AI tools for unintended consequences. **Infrastructure** (such as access, computing capacity and energy demand, and the human effort required to monitor AI tools) must be enhanced. Educational programs will incur upfront costs to develop AI capabilities that should ultimately provide a return on investment by saving precious faculty time and enhancing learning outcomes [65]. **Organizational culture** becomes even more important to stakeholder trust when gathering copious data about individuals and deploying AI supports. The AI use cases presented throughout this manuscript, while powerful for refining and personalizing competency development, imply a future state of nearly constant performance monitoring and feedback. To overcome the risk that learners will experience this well-intended process as a threatening surveillance state, AI-enabled educational systems must be deployed to the benefit of the learner and with the explicit involvement of learners in what is deployed and how it is deployed, remaining true to the learner-centered orientation at the heart of CBHPE [66,67]. The broader educational **environment** must offer guidance and resources. Accreditation, licensing, and continuing certification bodies must redesign their approaches to align with optimal use of AI.

Sociotechnical systems theory elevates key considerations for the successful application of AI to realize the vision of CBHPE. Applying STST in combination with change management frameworks could facilitate successful interventions and overcome implementation challenges [68]. Variations in health care systems, educational systems, and cultural contexts (both within a given country and across the international landscape) [69] will influence how readily AI can be integrated. Educators experimenting in this space should routinely engage multiple stakeholder groups to enhance project leaders’ contextual awareness. Transparency across institutions regarding both successes and struggles will be necessary to study how various dimensions of the system may enhance or inhibit the optimization of AI tools.

### Responsible application of AI in HPE

As in clinical applications of AI, there are critical elements to consider when leveraging AI tools in education. Educators must address issues such as data privacy and security, potential errors and bias in the data sets used to train algorithms and in the outputs of AI tools, and regulatory concerns. International guidelines should inform efforts [70,71]. To ensure responsible development and deployment of such tools, educators must pursue sufficient foundational training in AI to support their understanding of both its capabilities and limitations [2,72,73]. Educators are not expected to become experts in AI, but they must be sufficiently informed to ask appropriate questions of their more technical colleagues. Educators must demonstrate humility when applying novel AI approaches to CBHPE, remaining aware of limitations and risks such as premature labeling of learners or reinforcing narrow definitions of “good” performance. Transparency is important for building trust; learners should not only be informed about how AI contributes to their educational program and trained to contribute to continuous improvement but also be actively engaged in designing how AI achieves these goals in their training. Continuous monitoring of outcomes is imperative to reveal potential unintended consequences of AI use.

Intentional design for fairness and inclusivity, considering multiple stakeholders and settings, is necessary to support competency development. Variations in access to AI platforms, digital devices, and technical literacy among both learners and instructors may lead to discrepancies across individuals and clinical sites. Novel AI applications must be monitored for potential errors or bias in outputs. Well-funded and equipped academic and university medical centers are more likely to provide learners with AI-enabled educational innovations. In contrast, community hospitals or rural clinics may currently lack such technology [74]. Underfunded programs, including those in rural areas or community-focused schools, are more likely to encounter challenges in scaling or operationalizing such innovations [75,76]. The Irony of Need recognizes that lesser-resourced programs, typically serving populations with the most significant health disparities, face the greatest barriers in acquiring and implementing these innovations yet stand to benefit the most [77,78]. Innovators should strive to make tools as generalizable as possible where technically feasible. Impacts on the broader society and environment should be considered. The medical education community should collaborate to broaden AI’s reach in elevating competency among all health professionals.

## Conclusion

AI offers the potential to accelerate high-fidelity implementation of CBHPE across the health professions, positioning educators and programs to address long-standing challenges to realizing CBHPE’s core components. Sociotechnical systems theory provides pragmatic guidance for integrating AI, elevating the impact of incentives, workflows, and organizational culture on its effective incorporation. Educators must enhance their own understanding of AI and CBHPE to support responsible design and deployment of AI capabilities. With the rapidly evolving influence of AI across all aspects of education and healthcare, health professions educators must envision how health care will change over the coming decades in order to align education with needed competencies of the future. The health professions community must participate in a shared research agenda to highlight the many affordances of AI tools while promoting transparency around common challenges and errors encountered during experimentation and iteration.
